# 1,5-Dimethyl-4-[(5-methyl-2-furyl)­methyl­ene­amino]-2-phenyl-1*H*-pyrazol-3(2*H*)-one

**DOI:** 10.1107/S1600536808015419

**Published:** 2008-05-30

**Authors:** Yaning Guo

**Affiliations:** a Department of Chemistry, Baoji University of Arts and Science, Baoji, Shaanxi 721007, People’s Republic of China

## Abstract

In the title compound, C_17_H_17_N_3_O_2_, a derivative of 4-amino­anti­pyrine, the structure displays a *trans* configuration with respect to the imine C=N double bond. The pyrazoline ring is essentially planar and makes a dihedral angle of 55.80 (1)° with the phenyl ring.

## Related literature

For related literature, see: Ali *et al.* (2002 ▶); Allen *et al.* (1987 ▶); Carlton *et al.* (1995 ▶); Coolen *et al.* (1999 ▶); Cukurovali *et al.* (2002 ▶); Greisen & Andreasen (1976 ▶); Jiang *et al.* (2000 ▶); Liang *et al.* (2002 ▶); Tarafder *et al.* (2002 ▶).
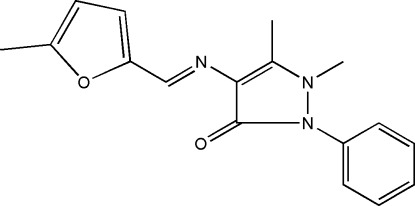


## Experimental

### 

#### Crystal data

C_17_H_17_N_3_O_2_
*M* *_r_* = 295.34
Monoclinic, 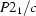

*a* = 11.811 (7) Å
*b* = 9.997 (6) Å
*c* = 14.116 (9) Å
β = 110.963 (9)°
*V* = 1556.4 (16) Å^3^
*Z* = 4
Mo *K*α radiation
μ = 0.09 mm^−1^
*T* = 296 K
0.40 × 0.40 × 0.40 mm

#### Data collection

Bruker SMART CCD area-detector diffractometer
Absorption correction: multi-scan (*SADABS*; Sheldrick, 2000 ▶) *T*_min_ = 0.967, *T*_max_ = 0.967
5012 measured reflections
2670 independent reflections
1904 reflections with *I* > 2σ(*I*)
*R*_int_ = 0.097

#### Refinement

*R*[*F* ^2^ > 2σ(*F* ^2^)] = 0.066
*wR*(*F* ^2^) = 0.154
*S* = 1.07
2670 reflections
203 parameters
H-atom parameters constrained
Δρ_max_ = 0.17 e Å^−3^
Δρ_min_ = −0.17 e Å^−3^

### 

Data collection: *SMART* (Bruker, 2000 ▶); cell refinement: *SAINT* (Bruker, 2000 ▶); data reduction: *SAINT*; program(s) used to solve structure: *SHELXS97* (Sheldrick, 2008 ▶); program(s) used to refine structure: *SHELXL97* (Sheldrick, 2008 ▶); molecular graphics: *SHELXTL* (Sheldrick, 2008 ▶); software used to prepare material for publication: *SHELXTL*.

## Supplementary Material

Crystal structure: contains datablocks I, global. DOI: 10.1107/S1600536808015419/bq2080sup1.cif

Structure factors: contains datablocks I. DOI: 10.1107/S1600536808015419/bq2080Isup2.hkl

Additional supplementary materials:  crystallographic information; 3D view; checkCIF report

## Figures and Tables

**Fig. 1.**
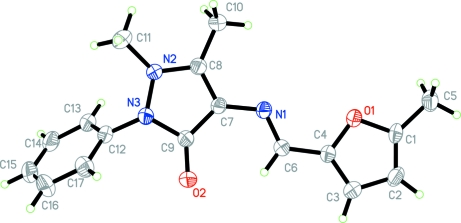
The structure of the title compound in 30% probability ellipsoids. H atoms are shown as spheres of arbitrary radii.

**Fig. 2.**
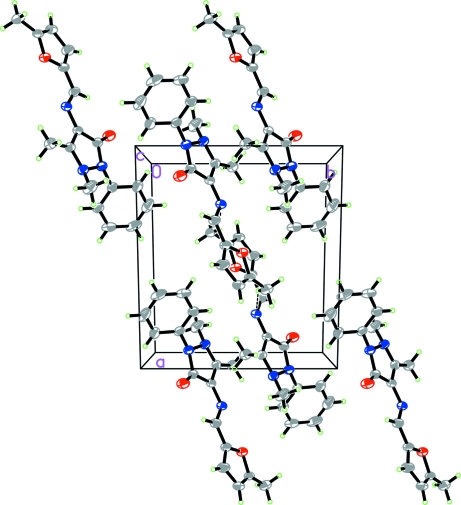
The molecular packing of (I) viewed along the b-axis.

## supplementary crystallographic information

### Comment

In recent years, the role of antipyrine and antipyridine derivatives in biological processes have become a topic of study (Carlton *et al.*, 1995; Coolen *et al.*, 1999; Jiang *et al.*, 2000). Antipyrine is an antipyretic drug that is still being used to measure the total hepatic oxidase activity. The properties of antipyrine make it a suitable marker for oxidative stress (Greisen & Andereasen, 1976). Schiff base ligands have demonstrated significant biological activities and new examples are being tested for their antitumor, antimicrobial and antiviral activities (Tarafder *et al.*, 2002; Cukurovali *et al.*, 2002; Ali *et al.*, 2002). These properties stimulated our interest in this field. Crystals of the title compound, (I), were obtained as a new antipyrine Schiff base compound.

The perspective view of the structure and a packing diagram of (I) are illustrated in Fig.1 and 2, respectively. All the bond lengths and angles are in normal ranges (Allen *et al.*, 1987) and comparable to those observed in a similar antipyrine Schiff base (Liang *et al.*, 2002). As seen from Fig. 1, the pyrazoline ring is essentially planar. Atom O2 deviates from the pyrazoline mean plane by -0.117 (5) Å, whereas atoms C10 and C11 by 0.115 (7) and 0.582 (7) Å, on the same side. The dihedral angle between the pyrazoline ring and the C12—C17 phenyl ring is 55.80 (1) °. The furan ring and the pyrazoline ring are approximately coplanar with the dihedral angle between them of 4.79 (2) °. As expected, the molecular structure adopts a *trans* configuration about the C6═N1 bond.

### Experimental

A mixture of 5-methyl-2-furaldehyd (0.1 mmol, 11.0 mg) and 4-aminoantipyrine (0.1 mmol, 20.3 mg) was dissolved in 10 ml methanol, and stirred for about 30 min at room temperature to give a clear yellow solution. After keeping this solution in air for 7 d, yellow block crystals were formed at the bottom of vessel by slowly evaporating the solvent.

### Refinement

All H atoms were placed in geometrically idealized positions and constrained to ride on their parent atoms with C—H distances in the range 0.93–0.96 Å and *U*_iso_(H) = 1.2*U*_eq_ or 1.5*U*_eq_(C/O)

### Crystal data

**Table:**

C_17_H_17_N_3_O_2_	*F*_000_ = 624
*M**_r_* = 295.34	*D*_x_ = 1.260 Mg m^−^^3^
Monoclinic, *P*2_1_/*c*	Mo *K*α radiation λ = 0.71073 Å
Hall symbol: -P 2ybc	Cell parameters from 3656 reflections
*a* = 11.811 (7) Å	θ = 2.6–27.1º
*b* = 9.997 (6) Å	µ = 0.09 mm^−^^1^
*c* = 14.116 (9) Å	*T* = 296 K
β = 110.963 (9)º	Block, yellow
*V* = 1556.4 (16) Å^3^	0.40 × 0.40 × 0.40 mm
*Z* = 4	

### Data collection

**Table:**

Bruker SMART CCD area-detector diffractometer	2670 independent reflections
Radiation source: fine-focus sealed tube	1904 reflections with *I* > 2σ(*I*)
Monochromator: graphite	*R*_int_ = 0.097
*T* = 296 K	θ_max_ = 25.0º
φ and ω scans	θ_min_ = 1.9º
Absorption correction: multi-scan(SADABS; Sheldrick, 2000)	*h* = −4→14
*T*_min_ = 0.967, *T*_max_ = 0.967	*k* = −11→9
5012 measured reflections	*l* = −16→16

### Refinement

**Table:**

Refinement on *F*^2^	Hydrogen site location: inferred from neighbouring sites
Least-squares matrix: full	H-atom parameters constrained
*R*[*F*^2^ > 2σ(*F*^2^)] = 0.066	*w* = 1/[σ^2^(*F*_o_^2^) + (0.0438*P*)^2^ + 0.5447*P*] where *P* = (*F*_o_^2^ + 2*F*_c_^2^)/3
*wR*(*F*^2^) = 0.154	(Δ/σ)_max_ < 0.001
*S* = 1.07	Δρ_max_ = 0.17 e Å^−^^3^
2670 reflections	Δρ_min_ = −0.17 e Å^−^^3^
203 parameters	Extinction correction: SHELXL97 (Sheldrick, 2008), Fc^*^=kFc[1+0.001xFc^2^λ^3^/sin(2θ)]^-1/4^
Primary atom site location: structure-invariant direct methods	Extinction coefficient: 0.048 (4)
Secondary atom site location: difference Fourier map	

### Special details

**Table:**

Geometry. All e.s.d.'s (except the e.s.d. in the dihedral angle between two l.s. planes) are estimated using the full covariance matrix. The cell e.s.d.'s are taken into account individually in the estimation of e.s.d.'s in distances, angles and torsion angles; correlations between e.s.d.'s in cell parameters are only used when they are defined by crystal symmetry. An approximate (isotropic) treatment of cell e.s.d.'s is used for estimating e.s.d.'s involving l.s. planes.
Refinement. Refinement of *F*^2^ against ALL reflections. The weighted *R*-factor *wR* and goodness of fit *S* are based on *F*^2^, conventional *R*-factors *R* are based on *F*, with *F* set to zero for negative *F*^2^. The threshold expression of *F*^2^ > σ(*F*^2^) is used only for calculating *R*-factors(gt) *etc*. and is not relevant to the choice of reflections for refinement. *R*-factors based on *F*^2^ are statistically about twice as large as those based on *F*, and *R*- factors based on ALL data will be even larger.

### Fractional atomic coordinates and isotropic or equivalent isotropic displacement parameters (Å^2^)

**Table:**

	*x*	*y*	*z*	*U*_iso_*/*U*_eq_	
O1	0.46426 (15)	−0.0177 (2)	0.11946 (13)	0.0512 (6)	
O2	0.10583 (17)	0.3027 (2)	0.15207 (15)	0.0696 (7)	
N1	0.23169 (19)	0.0950 (2)	0.05296 (15)	0.0455 (6)	
N2	−0.07371 (19)	0.1894 (2)	−0.08553 (15)	0.0486 (6)	
N3	−0.05032 (19)	0.2766 (3)	−0.00250 (16)	0.0519 (7)	
C7	0.1172 (2)	0.1547 (3)	0.01786 (18)	0.0433 (7)	
C1	0.5813 (2)	−0.0571 (3)	0.1754 (2)	0.0488 (7)	
C8	0.0317 (2)	0.1251 (3)	−0.07436 (18)	0.0449 (7)	
C10	0.0408 (3)	0.0357 (3)	−0.15564 (19)	0.0576 (8)	
H4A	0.0415	0.0887	−0.2121	0.086*	
H4B	−0.0275	−0.0238	−0.1773	0.086*	
H4C	0.1143	−0.0154	−0.1299	0.086*	
C6	0.3099 (2)	0.1276 (3)	0.1398 (2)	0.0547 (8)	
H5	0.2884	0.1906	0.1788	0.066*	
C9	0.0665 (2)	0.2508 (3)	0.0669 (2)	0.0486 (7)	
C2	0.6170 (3)	0.0008 (4)	0.2668 (2)	0.0621 (9)	
H7	0.6913	−0.0110	0.3191	0.075*	
C12	−0.1484 (3)	0.3267 (3)	0.02341 (19)	0.0511 (8)	
C4	0.4294 (2)	0.0702 (3)	0.1788 (2)	0.0525 (8)	
C13	−0.2464 (3)	0.2472 (4)	0.0175 (2)	0.0605 (9)	
H10	−0.2509	0.1595	−0.0054	0.073*	
C3	0.5208 (3)	0.0835 (4)	0.2690 (2)	0.0704 (10)	
H11	0.5206	0.1371	0.3227	0.084*	
C15	−0.3310 (4)	0.4306 (5)	0.0795 (3)	0.0885 (14)	
H12	−0.3929	0.4662	0.0978	0.106*	
C17	−0.1411 (3)	0.4580 (4)	0.0576 (2)	0.0673 (9)	
H13	−0.0750	0.5113	0.0615	0.081*	
C5	0.6407 (3)	−0.1481 (4)	0.1252 (2)	0.0652 (9)	
H14A	0.7193	−0.1726	0.1722	0.098*	
H14B	0.6492	−0.1038	0.0678	0.098*	
H14C	0.5921	−0.2270	0.1029	0.098*	
C11	−0.1602 (3)	0.2351 (4)	−0.1828 (2)	0.0852 (13)	
H15A	−0.1321	0.3176	−0.2015	0.128*	
H15B	−0.2378	0.2492	−0.1769	0.128*	
H15C	−0.1675	0.1688	−0.2338	0.128*	
C14	−0.3378 (3)	0.2996 (5)	0.0461 (2)	0.0763 (11)	
H16	−0.4039	0.2468	0.0429	0.092*	
C16	−0.2332 (4)	0.5077 (4)	0.0854 (3)	0.0879 (13)	
H17	−0.2288	0.5953	0.1086	0.105*	

### Atomic displacement parameters (Å^2^)

**Table:**

	*U*^11^	*U*^22^	*U*^33^	*U*^12^	*U*^13^	*U*^23^
O1	0.0387 (11)	0.0633 (14)	0.0496 (10)	0.0038 (10)	0.0134 (8)	−0.0004 (10)
O2	0.0511 (13)	0.0848 (17)	0.0624 (13)	0.0078 (12)	0.0077 (10)	−0.0311 (12)
N1	0.0359 (13)	0.0542 (16)	0.0473 (12)	−0.0030 (11)	0.0159 (10)	−0.0022 (11)
N2	0.0441 (14)	0.0550 (16)	0.0423 (12)	0.0061 (12)	0.0099 (10)	−0.0064 (11)
N3	0.0401 (13)	0.0561 (16)	0.0545 (13)	0.0049 (12)	0.0106 (10)	−0.0120 (12)
C7	0.0361 (15)	0.0492 (18)	0.0442 (14)	−0.0031 (13)	0.0139 (11)	−0.0025 (13)
C1	0.0309 (15)	0.057 (2)	0.0583 (16)	0.0012 (13)	0.0153 (12)	0.0114 (14)
C8	0.0445 (16)	0.0485 (18)	0.0441 (14)	−0.0002 (14)	0.0186 (12)	0.0029 (13)
C10	0.0570 (19)	0.067 (2)	0.0473 (15)	0.0056 (17)	0.0167 (13)	−0.0030 (15)
C6	0.0446 (17)	0.065 (2)	0.0531 (16)	0.0048 (15)	0.0158 (13)	−0.0141 (15)
C9	0.0405 (16)	0.0509 (18)	0.0517 (15)	0.0003 (14)	0.0130 (12)	−0.0094 (14)
C2	0.0388 (16)	0.073 (2)	0.0631 (18)	−0.0015 (17)	0.0039 (13)	−0.0026 (17)
C12	0.0445 (17)	0.054 (2)	0.0485 (15)	0.0101 (15)	0.0083 (12)	0.0002 (14)
C4	0.0373 (16)	0.062 (2)	0.0562 (16)	−0.0004 (14)	0.0146 (13)	−0.0091 (15)
C13	0.0462 (18)	0.067 (2)	0.0596 (17)	0.0060 (17)	0.0080 (14)	−0.0076 (16)
C3	0.0514 (19)	0.086 (3)	0.0624 (18)	0.0040 (19)	0.0064 (15)	−0.0222 (18)
C15	0.060 (2)	0.128 (4)	0.076 (2)	0.039 (3)	0.0220 (18)	−0.011 (2)
C17	0.068 (2)	0.055 (2)	0.076 (2)	0.0100 (18)	0.0231 (17)	−0.0030 (17)
C5	0.0510 (18)	0.081 (3)	0.0661 (18)	0.0154 (18)	0.0237 (15)	0.0138 (18)
C11	0.084 (2)	0.101 (3)	0.0503 (17)	0.032 (2)	−0.0009 (17)	−0.0012 (19)
C14	0.0406 (19)	0.113 (4)	0.069 (2)	0.010 (2)	0.0120 (15)	−0.005 (2)
C16	0.097 (3)	0.075 (3)	0.090 (3)	0.035 (3)	0.030 (2)	−0.009 (2)

### Geometric parameters (Å, °)

**Table:**

O1—C4	1.375 (3)	C2—H7	0.9300
O1—C1	1.382 (3)	C12—C13	1.382 (4)
O2—C9	1.237 (3)	C12—C17	1.390 (4)
N1—C6	1.285 (3)	C4—C3	1.349 (4)
N1—C7	1.397 (3)	C13—C14	1.383 (5)
N2—C8	1.359 (3)	C13—H10	0.9300
N2—N3	1.407 (3)	C3—H11	0.9300
N2—C11	1.461 (3)	C15—C16	1.366 (6)
N3—C9	1.401 (3)	C15—C14	1.384 (6)
N3—C12	1.424 (4)	C15—H12	0.9300
C7—C8	1.364 (3)	C17—C16	1.375 (5)
C7—C9	1.435 (4)	C17—H13	0.9300
C1—C2	1.337 (4)	C5—H14A	0.9600
C1—C5	1.475 (4)	C5—H14B	0.9600
C8—C10	1.488 (4)	C5—H14C	0.9600
C10—H4A	0.9600	C11—H15A	0.9600
C10—H4B	0.9600	C11—H15B	0.9600
C10—H4C	0.9600	C11—H15C	0.9600
C6—C4	1.438 (4)	C14—H16	0.9300
C6—H5	0.9300	C16—H17	0.9300
C2—C3	1.414 (4)		
			
C4—O1—C1	106.9 (2)	C17—C12—N3	117.8 (3)
C6—N1—C7	120.3 (2)	C3—C4—O1	109.0 (3)
C8—N2—N3	107.33 (19)	C3—C4—C6	131.8 (3)
C8—N2—C11	124.1 (2)	O1—C4—C6	119.2 (2)
N3—N2—C11	116.8 (2)	C12—C13—C14	119.3 (3)
C9—N3—N2	108.6 (2)	C12—C13—H10	120.4
C9—N3—C12	124.9 (2)	C14—C13—H10	120.4
N2—N3—C12	119.8 (2)	C4—C3—C2	107.5 (3)
C8—C7—N1	122.5 (2)	C4—C3—H11	126.3
C8—C7—C9	108.2 (2)	C2—C3—H11	126.3
N1—C7—C9	129.3 (2)	C16—C15—C14	120.0 (4)
C2—C1—O1	109.5 (3)	C16—C15—H12	120.0
C2—C1—C5	133.7 (3)	C14—C15—H12	120.0
O1—C1—C5	116.8 (2)	C16—C17—C12	118.9 (4)
N2—C8—C7	110.0 (2)	C16—C17—H13	120.5
N2—C8—C10	120.7 (2)	C12—C17—H13	120.5
C7—C8—C10	129.3 (3)	C1—C5—H14A	109.5
C8—C10—H4A	109.5	C1—C5—H14B	109.5
C8—C10—H4B	109.5	H14A—C5—H14B	109.5
H4A—C10—H4B	109.5	C1—C5—H14C	109.5
C8—C10—H4C	109.5	H14A—C5—H14C	109.5
H4A—C10—H4C	109.5	H14B—C5—H14C	109.5
H4B—C10—H4C	109.5	N2—C11—H15A	109.5
N1—C6—C4	122.4 (3)	N2—C11—H15B	109.5
N1—C6—H5	118.8	H15A—C11—H15B	109.5
C4—C6—H5	118.8	N2—C11—H15C	109.5
O2—C9—N3	122.4 (3)	H15A—C11—H15C	109.5
O2—C9—C7	132.3 (2)	H15B—C11—H15C	109.5
N3—C9—C7	105.2 (2)	C13—C14—C15	120.1 (4)
C1—C2—C3	107.2 (3)	C13—C14—H16	120.0
C1—C2—H7	126.4	C15—C14—H16	120.0
C3—C2—H7	126.4	C15—C16—C17	121.1 (4)
C13—C12—C17	120.7 (3)	C15—C16—H17	119.5
C13—C12—N3	121.6 (3)	C17—C16—H17	119.5
